# Sphingosine 1-Phosphate signaling controls mitosis

**DOI:** 10.18632/oncotarget.22310

**Published:** 2017-11-07

**Authors:** Olivier Cuvillier, Anastassia Hatzoglou

**Affiliations:** Institut de Pharmacologie et de Biologie Structurale, IPBS,Université de Toulouse, CNRS, UPS, Toulouse, France

**Keywords:** mitosis, sphingosine-1phosphate, sphingosine kinase, S1P_5_, cell signaling

Besides their structural role, sphingolipids including Sphingosine-1phosphate (S1P) have drawn attention as bioactive sphingolipid signaling molecules involved in the regulation of several processes, including cell growth, differentiation, survival, migration or immune response. It is now well established that S1P is involved in the onset or the progression of pathological conditions such as cancer, autoimmunity, cardiovascular conditions or diabetes to name a few. The S1P content in cells is low and is kept under control through a delicately regulated balance between its synthesis by Sphingosine kinases 1 & 2 (SphKs) and its dephosphorylation by S1P phosphatases or degradation by S1P lyase. Once produced S1P can be secreted via specific ATP-binding cassette transporters including Spns2 and exerts its extracellular functions through a family of five G-protein-coupled receptors, named S1P_1-5_. Thus, this *inside-out* signaling is critical for a variety of S1P cellular responses. Deregulation of S1P metabolism has been related to various diseases including cancer with increased S1P tissue levels. Various studies suggest the involvement of SphKs/S1P signaling in tumor progression and metastasis [1, 2].

Cell division or mitosis is a particularly complex and highly regulated process that allows the formation of two genetically identical daughter cells. A quality control mechanism called spindle assembly checkpoint (SAC, sometimes referred as mitotic checkpoint), ensures fidelity of chromosome segregation. The SAC prevents anaphase onset until all kinetochores are stably attached to microtubules. In the presence of unattached kinetochore, the SAC is on and the metaphase-to-anaphase transition is inhibited. Dysfunction of SAC leads to chromosome mis-segregation and aneuploidy and is implicated in tumorigenesis [3, 4].

We recently identified and explored a novel function of SphKs/S1P signaling during mitosis [5]. Our study began with the observation that SphK1 silencing increased the mitotic rate whereas its ectopic expression decreased it. Detailed analysis revealed a SAC-dependent mitotic delay before metaphase in cells lacking S1P. Pharmacological inhibition of SphK1 led to mitotic delay similar to SphK1 silencing indicating that mitotic function of SphK1 is related to its enzymatic activity. The prediction was that SphK1 controls mitosis through its product S1P. Indeed, cells treated with S1P, completed mitosis much faster. S1P induced SAC relaxation leading to chromosome segregation defects. However, understanding the pathophysiological consequences of S1P-induced chromosome segregation defects will require further studies.

S1P may act intra- or extracellularly. To test this hypothesis we used Sphingomab™, a high-affinity monoclonal anti-S1P antibody that neutralizes extracellular S1P [6]. First, Sphingomab completely blocked mitotic acceleration induced by both S1P and SphK1 overexpression. Second, silencing Spns2, the major S1P transporter, blocked S1P release and resulted in mitotic delay. Third, conditioned media from prostate cancer PC3 cells, that produce and secrete high amounts of S1P, accelerated mitosis, a paracrine effect that could be prevented by the anti-S1P antibody. Thus, S1P is secreted through the transporter Spns2 and stimulates mitosis in auto/paracrine manner.

**Figure 1:**
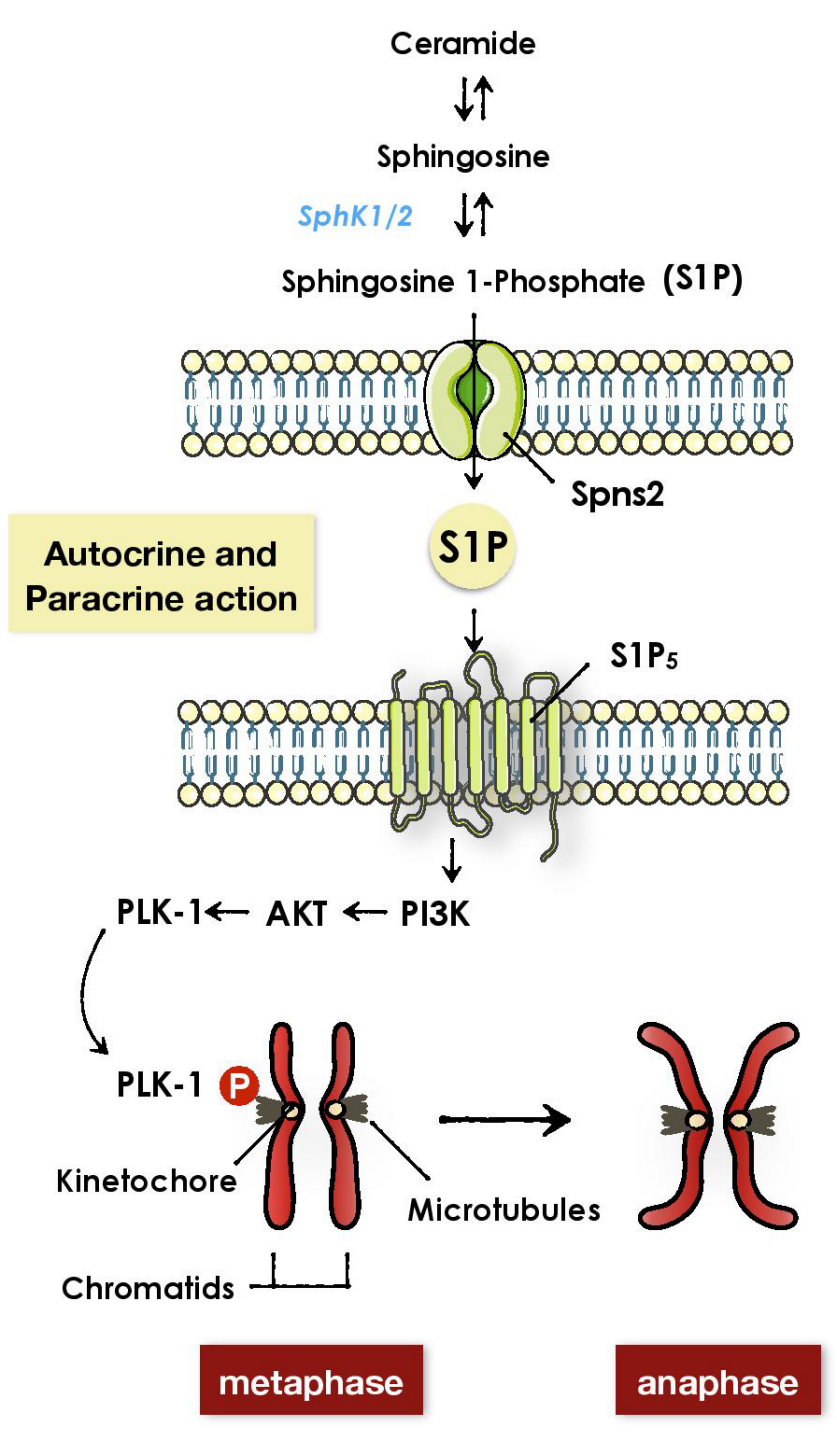
The role of S1P/S1P_5_ pathway in mitotic regulation

Five high-affinity G-coupled receptors (S1P_1-5_) mediate S1P extracellular functions. These receptors differ in their tissue distribution, and their biological effect, depending on the suite of S1P receptor subtypes expressed. Using pharmacological and RNA interference approaches we identified S1P_5_ receptor as target for S1P’s mitotic function. Accordingly, mitosis was not affected by S1P in mouse embryonic fibroblasts (MEFs) derived from S1P_5_ knockout mice. Among multiple signaling cascades activated downstream S1P receptors, PI3K/Akt pathway was activated by S1P in mitotic cells. It has been reported that Akt phosphorylates and activates the mitotic kinase Polo-like kinase 1 (Plk1) at Serine 99 at the level of kinetochore and promotes metaphase-to-anaphase transition [7]. Using Tet-ON HeLa cell lines expressing wild type (wt) or mutant (S99A) PLK1, we showed that S1P-induced mitotic phenotype requires phosphorylation of Plk1 at Ser^99^. Therefore, abundance of S1P promotes mitotic progression by S1P_5_ and downstream activation of PI3K/Akt leading to Plk1 activation at kinetochore, to control metaphase-to-anaphase transition (Figure 1).

Overall, these data demonstrate that extracellular S1P promotes mitotic progression leading to chromosome segregation defects supporting the concept that cellular microenvironment plays an important role in coordination of mitosis. In the future, it will be important to investigate what are the consequences of the S1P-induced chromosome mis-segregation in chromosome stability of normal and cancer cells and the therapeutic potential of S1P/S1P_5_ axis.
